# Mania as a Rare Adverse Event Secondary to Steroid Eye Drops

**DOI:** 10.1155/2022/4456716

**Published:** 2022-06-16

**Authors:** Moayyad Alsalem, Majed A. Alharbi, Rayan A. Alshareef, Raghad Khorshid, Salman Thabet, Abdulrahman Alghamdi

**Affiliations:** ^1^Psychiatry Section, Department of Medicine, King Abdulaziz Medical City, Ministry of the National Guard-Health Affairs, Jeddah, Saudi Arabia; ^2^King Abdullah International Medical Research Center, Jeddah, Saudi Arabia; ^3^College of Medicine, King Saud Bin Abdulaziz University for Health Sciences, Jeddah, Saudi Arabia; ^4^Department of Adult Mental Health, King Abdulaziz Medical City, Ministry of the National Guard-Health Affairs, Riyadh, Saudi Arabia; ^5^King Abdullah International Medical Research Center, Riyadh, Saudi Arabia; ^6^Department of Ophthalmology, King Abdulaziz University, Jeddah, Saudi Arabia

## Abstract

Since glucocorticoids (GCs) were introduced for the treatment of various diseases, they have been linked with the development of psychiatric adverse effects such as mania, depression, and psychosis. These behavioral or psychiatric adverse events usually appear within a few days after commencing GCs and are possibly to reverse with drug withdrawal. We present a rare case of a 75-year-old woman who developed mania during treatment with GC eye drops following cataract surgery. Management consisted of discontinuing prednisolone and administering olanzapine, which resulted in full recovery in a week. Olanzapine was then discontinued, and a diagnosis of steroid-induced mania was concluded for this case.

## 1. Introduction

Since the glucocorticoids (GCs) were introduced in the 1950s, they have been proven effective in treating various systemic diseases including acute and chronic allergic and inflammatory conditions [1]. However, GCs were also known to cause multiple behavioral and psychiatric adverse events such as mania, psychosis, depression, anxiety, and cognitive decline [2]. Symptoms can appear after short- or long-term use at any time during the treatment course, most often within a few days after initiation [3]. These adverse events are usually reversible with discontinuation of medication; however, the additional use of psychotropic medication may be warranted [3]. Here, we present a case of an elderly woman who developed mania following starting GC eye drops postcataract surgery. Informed consent was obtained for this case report.

## 2. Case Presentation

A 75-year-old woman who had cataract started using prednisolone 1% eye drops four times a day postoperatively. She presented to the psychiatry outpatient clinic nine days after surgery, with a seven-day history of elated mood, pressured speech, flight of ideas, insomnia, irritability, and restlessness. She had a decreased need for sleep (3–4 hours of sleep per night) compared to a baseline sleep of 7 hours per night. Her motor and verbal activity increased markedly. She displayed restlessness, talkativeness, and irritability during the clinical interview. In her psychiatric assessment, she was cooperative and oriented to time, place, and the people around her. She did not show grandiosity or report delusions, but she admitted experiencing visual hallucinations. She had no history of any substance use and no known drug allergy. According to her family, her psychiatric manifestations started two days after the initiation of the prednisolone eye drops.

After a thorough review of her medications, the possible causal relationship between the prednisolone eye drops and manic episode was suspected. The Young Mania Rating Scale (YMRS) score was 29 out of 60 [4]. Complete physical examination and thorough laboratory investigations including full blood cell count, liver, renal, and thyroid function tests, and vitamin levels were noncontributory. After consultation with her ophthalmologist, the prednisolone was discontinued, and olanzapine orally disintegrating tablet (5 mg/day at bedtime) was given for her insomnia and agitation. The patient's behavior improved rapidly over a week. The YMRS score decreased to 6 out of 60. She was followed for 3 months and remained stable and without any psychiatric manifestations. Olanzapine was discontinued after 2 months.

According to the clinical picture and history with a clear temporal association between the use of the steroid eye drops and the onset of manic symptoms, a diagnosis of steroid-induced mania was reached.

## 3. Discussion

Steroid-induced psychiatric symptoms are well-documented phenomena and are typically seen with high doses and systemic administration of corticosteroids [1]. However, less is known about the psychiatric adverse events of topically administered medications. They are inevitably absorbed from the eye into the systemic circulation and thus have the potential to cause systemic adverse effects like mania and acute confusion [5]. The reported case emphasizes that even a drug that is administered topically can cause adverse psychiatric effects. To our knowledge, only four cases were reported in the literature for neuropsychiatric manifestations shortly after starting GC eye drops (Table 1) [6–9].

Most psychiatric symptoms begin within a few days of GC treatment [3]. In our case, manic symptoms developed on the second day of steroid treatment. The literature on the treatment of steroid-induced mania is limited to only case reports. A suggested management may start with GS eye drop dose reduction or cessation. In some cases, psychotropic medications may be warranted due to the inability to discontinue steroid treatment or the severity of psychiatric symptoms [3]. In this case, we discontinued the steroid therapy and added olanzapine for the patient's insomnia and psychomotor agitation. One only of the five reported cases, including ours, was successful with only stopping the offending agent without adding a psychotropic medication.

Clinicians should consider the adverse psychiatric effects of topical corticosteroids, especially in the elderly population, and inform patients about them. Awareness about this rare occurrence should be raised to allow for early monitoring and for more cases to be reported to help guide prevention efforts and clinical decision-making about the proper management of steroid-induced mania.

## Figures and Tables

**Table 1:** Summary of reported cases of neuropsychiatric adverse effects associated with glucocorticoid eye drop use.

Authors, year	Age in years (sex)	Type of glucocorticoid eye drops (dose)	Past psychiatric history	Neuropsychiatric manifestations	Management	Outcome
Mok and Malladi, 2013 [6]	81 (female)	Prednisolone (1% four times/day)	Chronic schizophrenia and single episode of hypomania	Mania	(i) Prednisolone discontinued (ii) Uptitration of baseline psychotropic medications	Full recovery
Kumagai and Ichimiya, 2014 [7]	76 (male)	Fluorometholone (N/A)	N/A	Mania	(i) Fluorometholone discontinued (ii) Sodium valproate (200 mg/day) administered	Full recovery
Farooq and Dallol, 2014 [8]	90 (female)	Fluorometholone (0.1% three times/day)	None	Acute confusion	(i) Fluorometholone discontinued	Full recovery
Cakici and Hergüner, 2015 [9]	15 (male)	Fluorometholone (0.1% three times/day)	Attention-deficit/hyperactivity disorder	Hypomania	(i) Fluorometholone discontinued (ii) Quetiapine (100 mg/day) administered	Full recovery
This case	75 (female)	Prednisolone (1% four times/day)	None	Mania	(i) Prednisolone discontinued (ii) Olanzapine (5 mg/day) administered	Full recovery

N/A: not available.
